# REM sleep latency and neurocognitive dysfunction in schizophrenia

**DOI:** 10.4103/0019-5545.55934

**Published:** 2005

**Authors:** Mrinmay Das, Ruchika Das, Udayan Khastgir, Utpal Goswami

**Affiliations:** * Consultant Psychiatrist, Department of Psychiatry and Drug De-addiction Centre, Lady Hardinge Medical College and Associated Hospitals, New Delhi 110001; ** Clinical Psychologist, Department of Psychiatry and Drug De-addiction Centre, Lady Hardinge Medical College and Associated Hospitals, New Delhi 110001; *** Consultant Psychiatrist, Department of Psychiatry and Drug De-addiction Centre, Lady Hardinge Medical College and Associated Hospitals, New Delhi 110001; **** Ex-Professor and Head, Department of Psychiatry and Drug De-addiction Centre, Lady Hardinge Medical College and Associated Hospitals, New Delhi 110001

**Keywords:** REM sleep latency, neurocognitive deficits, negative symptoms, schizophrenia

## Abstract

**Background:**

Cognitive deficits—the hallmark of schizophrenic deterioration—still remain elusive as far as their pathophysiology is concerned. Various neurotransmitter systems have been implicated to explain these deficits. Abnormalities in cholinergic neurotransmission in the brain are one of the postulations; acetylcholine has also been postulated to regulate rapid eye movement (REM) sleep, especially REM latency. Thus, REM latency in patients with schizophrenia might provide a non-invasive window to look into the cholinergic functions of the brain.

**Aim:**

To study REM sleep measures and neurocognitive function in schizophrenia, and the changes occurring in these parameters following pharmacological treatment.

**Methods:**

Thirty subjects (15 with schizophrenia and 15 normal non-relative controls) were evaluated in this study. Most patients with schizophrenia had prominent negative symptoms and deficits in the performance in neurocognitive tests battery. They were treated with antipsychotics for a variable period of time and post-treatment evaluation was done using the same battery of neurocognitive tests and polysomnography. Patients were either drug-naïve or kept drug-free for at least two weeks both at baseline as well as at the post-treatment stage.

**Results:**

A positive correlation between the severity of negative symptoms and neurocognitive deficits (especially on the Wisconsin Card Sorting), and a negative correlation between these two parameters and REM latency was observed.

**Conclusion:**

It can be hypothesized that the acetylcholine deficit model of dementia cannot be applied to schizophrenic dementia, rather a hypercholinergic state results. This state warrants anticholinergic medication as a treatment option for negative symptoms of schizophrenia.

## INTRODUCTION

Cognitive impairment is a central manifestation of schizophrenia.^1^,^2^ It impacts on the quality of life of patients and on the cost of illness to society.^3^ In recent years, researchers have attempted to identify and quantify the deficits through neuropsychological, neurophysiological and imaging studies of the brain; they have tried to identify specific regional brain abnormalities corresponding to these deficits. Using neuropsychological tests, some investigators have reported relatively greater left hemispheric deficits among patients with schizophrenia.^4^,^5^ Others have found a more generalized and non-specific pattern of neurocognitive dysfunction.^6^ Many investigators have focused on neuropsychological evidence of frontal lobe dysfunction in schizophrenia, and some supportive evidence has been reported.^7^,^8^ Cerebral blood flow studies^9^ and positron emission tomographic investigations^10^ have provided further evidence of frontal lobe dysfunction in schizophrenia. More recently, neuroradiological metabolic studies have hypothesized abnormal connectivity between the frontal lobe and temporal areas, and have suggested an association between these functional abnormalities and cognitive impairment.^11^ Despite these attempts, defining neurocognitive deficits along biological lines have remained elusive.^12^

As the pathophysiology of cognitive impairment in schizophrenia is unknown, no rational pharmacotherapy for this condition can be devised. Despite the fact that patients with schizophrenia do not show cholinergic deficits, drawing a similarity between Alzheimer disease and neurocognitive deterioration in schizophrenia, cholinomimetics might be tried to improve cognitive dysfunction in schizophrenia.^13^

On the other hand, it has been shown that shortened rapid eye movement (REM) latency is a non-specific finding, is present in depressive illness, and correlates negatively with the severity of negative symptoms of schizophrenia.^14^,^15^ Cholinomimetic drugs have been used to show their REM latency-reducing property, with the hypothesis that increased cholinergic function or decreased aminergic function or both might be responsible for shortened REM latency. Here lies the paradox that cognitive impairment (hypocholinergic state), shortened REM latency and increased severity of negative symptoms (hypercholinergic state) are present in the same patient and at the same time. To understand the paradox, we measured the REM latency vis-a-vis negative symptoms and cognitive function.

We studied REM sleep measures and neurocognitive functions in schizophrenia. The changes occurring in these parameters following pharmacological treatment were also studied.

## METHODS

### Subjects

There were a total of 30 subjects (15 cases with schizophrenia and 15 normal controls) in the current investigation. All the patients with schizophrenia were recruited from among those seeking treatment at the OPD and inpatient services of the Department of Psychiatry, Lady Hardinge Medical College and Associated Hospitals, New Delhi. The clinical staff of the department had been told that the investigators were seeking previously unmedicated (drug-naive) or currently medication-free (for at least two weeks) patients with schizophrenia and were asked to contact the investigators. Given the rarity of such subjects, continuous surveillance of inpatient admissions was also maintained.

The patients being considered for this study were interviewed to determine whether they met the DSM-IIIR^16^ criteria for chronic schizophrenia, as confirmed by the Structured Clinical Interview for DSM-IIR, Patient version (SCID-P).^17^ The interview was supplemented by a review of the patients' records and an interview of family members. Patients who did not meet the inclusion criteria or met the exclusion criteria (Box 1) were kept out of the study. The patients we studied were chronically disabled but did not need acute hospitalization.

**Box 1:** Exclusion criteria for selection of schizophrenics

History of or presence of current organic diseases History of alcohol or other substance abuse or dependence Patients with epilepsy or mental retardation Patients with serious suicidal or homicidal potential Patients with primary neurological disorders or any other Axis 1 disorder apart from schizophrenia Patients with primary sleep disorders

Informed consent was taken from the subjects who were able and willing; alternatively, if the subjects were judged to be incompetent to give written informed consent, the same was obtained from significant relatives.

A battery of relevant investigations was done to rule out any other medical, surgical or neurological disorder. A semi-structured proforma devised for the study was used to collect sociodemographic information and historical data, with special reference to clinico-symptomatological, course-related and treatment outcome variables, including data on standard physical and detailed mental status examination.

Over a period of 13 months, a total of 23 drug-naive or psychoactive medication-free (for at least two weeks) patients were approached to participate in this study. Two patients refused. Four patients required medication to control agitation before they could be studied and hence were excluded. A seventh patient was excluded as his illness turned out to be schizoaffective disorder. Thus, of the original cohort of 23, 15 patients fulfilled the inclusion and exclusion criteria and were included in the study.

Subjects in the control group were selected from the clinical/paraclinical staff members of the department as well as healthy attendants who were not biologically related to the inpatients. The age, sex and educational status of the control group were matched as closely as possible with that of the index group (Table 1).

**Table 1:** Sociodemographic and clinical profile of the study groups

Variable	Index cases	Normal controls
Age (in years; mean±SD)	32.1±6.7	31±8.1
Sex (M/F)	8/7	8/7
Duration of illness (in years; mean±SD)	4.6±4.1	0
Course of illness		
Chronic–stable	3	—
Waxing–waning	8	—
Progressively deteriorating	4	—
Subtypes		
Paranoid	9	—
Residual	3	—
Undifferentiated	3	—
Duration of medication (in months; mean±SD)	6.3±2.4	—

The mean age of the patient group was 32.1±6.7 years (range: 22–46 years) and that of the control group 31.0±8.1 years (range: 19–45 years). The majority of the subjects (both cases and controls) were educated till higher secondary (10+2), without any skew between the grouping, and the groups were comparable.

The average duration of illness was 4.6±4.1 years; 60% of the cases were diagnosed with paranoid, and 20% each with residual and undifferentiated schizophrenia. Of the 15 cases, 3 patients were drug-naive, 2 had been medicated for 1 year, 2 for 6 months, 2 for 3 months, 3 for 5 weeks and 3 for 2 weeks. Therefore, the mean duration of medication was 6.3±2.4 months.

The Positive and Negative Syndrome Scale (PANSS),^18^ Montgomery–Asberg Depression Rating Scale (MADRS),^19^ Abnormal Involuntary Movement Scale (AIMS),^20^ Simpso–Angus rating scale (to measure extrapyramidal side-effects)^21^ were administered to each subject to obtain a set of baseline measures in keeping with the aim of the study. The Global Assessment of Functioning (GAF) Scale of DSM-IV^22^ was applied to assess the current level of global functioning.

### REM sleep study procedure

Polysomnography was conducted on two consecutive nights in the sleep laboratory. Recording on the first night allowed acclimatization of the subject to the sleep laboratory environment. The polygraphic data of only the second night were used in the analysis.

Sleep was recorded in the laboratory on a 24-channel polygraph (MPA-S Medilog instruments, Oxford). An electroencephalogram (EEG), an electro-oculogram (EOG) and a bipolar submental chin electromyogram (EMG) recorded the sleep state. The EEG consisted of a C4-scalp placement referenced to linked mastoids. The EOG consisted of electrode placement at the outer canthi of the eye with the derivation of each eye also referenced to the linked mastoids. All electrode impedances were determined to be less than 5000 ohms. The filter setting was 1–30 Hz for the EEG, 0.3–30 Hz for the EOG and 10–90 Hz for the submental chin EMG.

All sleep recordings were scored in 30 second epochs following Rechtschaffen–Kales criteria.^23^ Sleep onset was defined by the first minute of 10 consecutive minutes of stage II non-rapid eye movement (NREM) sleep, with no more than 2 intervening minutes of stage I sleep or awakening, following lights out.

REM sleep was defined as not less than 3 consecutive minutes of REM sleep, with no less than 20 minutes of NREM sleep (stages I through IV) subtending any two REM periods. Sleep architecture was defined in terms of percentage of sleep time spent in NREM stages I through IV and REM.

### Assessment of cognitive functions

To test cognitive functions, a battery of tests was applied by means of a computer-assisted NeuroScan machine utilizing STIM software (Neurosoft Inc. 1990, STIM Audio system). The following tests were used: Card Sort, Visual Continuous Performance Task (VISCPT), Stroop and Spatial Task.

The Card Sort program has been modelled after the Wisconsin Card Sorting Test (WCST),^24^,^25^ which was designed to test ‘abstract behaviour’ and ‘shift of set’. The program presents the subjects with a set of four card-like stimuli with a different pattern on each. The patterns contain different shapes in different colours. A probe stimulus is presented in the lower portion of the screen. The subject was required to choose the card from among the original four that best matched the probe stimulus. There were three randomly varied response contingencies. First, the subjects might be required to respond on the basis of the colour of the image on the card. Second, the subjects might be required to respond on the basis of the pattern of the image. Third, the subjects might be required to respond on the basis of the number of images on the card. The patients were not informed of the correct principle but a feedback on whether they were correct or not was given after their matching of each card. Once the criterion of 10 correctly sorted cards is attained, the principle is changed; the patient is not informed of this change. The test proceeds until the patient has completed six sorting categories, each consisting of 10 consecutive cards. The types of errors that are elicited may vary, although the most sensitive response type with respect to frontal lobe dysfunction is the perseverative response, reflecting subjects' difficulty in shifting their strategies or cognitive sets.

The Visual Continuous Performance Task (VISCPT) was used to test the attention span. In this program, user-created visual stimuli which could be precisely timed at rates were presented on the computer screen. The subject had to press the left button of the mouse when a ‘0’ appeared and the right button for all other digits that appeared on the screen.

Spatial Task was used to test spatial memory. In this test, the number of object locations, and the time between stimulus element presentation and response could be varied. The scoring percentage of right/wrong among the total number of stimuli presented tested the spatial memory.

Stroop Test^26^ was used as a measure of reading fluency and frontal lobe dysfunction. This task required subjects to rapidly shift the perceptual set when viewing names of colours that appeared in matching or non-matching colours. The Stroop program allowed the presentation of up to four different words in four different colours (e.g. red, yellow, green and blue). The program randomly presented the words in congruent and incongruent colours.

The patients were first familiarized with the tests and were then helped to give the test on their own, after which the data were taken for analysis.

Following the baseline assessments, patients in the schizophrenic group were allocated to either the haloperidol or risperidone group. Seven patients were treated with haloperidol as the main antipsychotic and the remaining 8 with risperidone for a variable period, ranging from 4 weeks to 14 weeks.

Next, various measures including symptom profile, side-effects profile, level of functioning, sleep polygraph measures were repeated for the entire cohort of patients with schizophrenia keeping them off medications for two weeks before the test.

The normal healthy controls also underwent the same evaluations, including a polysomnography study and neurocognitive assessment, but only once.

## RESULTS

### Clinical profile of the patients

Table 2 shows the mean psychopathology scores in the index group, both before and after treatment with antipsychotics. All the three subscale scores of the index group (positive, negative and general psychopathology subscales in PANSS) were significantly more than those of the control group (significant at p<0.05). These scores remained significantly higher before and after the treatment. In the index group, there were significant changes in the positive and negative subscale scores after treatment (19.1±6.4 to 8.3±1.3, p=0.03 and 27±6.3 to 16.1±5.0, p=0.003, respectively); however, the change in the general psychopathology score was not significant (40.6±4.4 to 22.4±2.9, p=0.06).

**Table 2:** Psychopathology scores of the index group

	Score (mean±SD)	
	
Parameter	Before treatment	After treatment
PANSS		
Positive subscale	19.1±6.4	8.3±1.3
Negative subscale	27.0±6.3	16.1±5.0
General psychopathology subscale	40.6±4.4	22.4±2.9
MADRS	18.6±7.2	11.6±4.8
AIMS*		
Simpson–Angus rating scale (for extrapyramidal symptoms) score†		

PANSS: Positive and Negative Syndrome Scale; MADRS: Montgomery–Asberg Depression Rating Scale; AIMS: Abnormal Involuntary Movement Scale

* Three out of 15 patients had been suffering from mild to moderately severe tardive dyskinesia which neither deteriorated nor improved with treatment of short duration.

† Among the experimental group, 11 patients developed antipsychotic-induced minimal to mild extrapyramidal symptoms that were significantly more than in the control group.

Table 3 shows that there was a significant decrease in REM latency in the patient group before treatment (61.4±34.7 minutes in the index group and 100.6±20.2 minutes in the control group, p=0.001). In the post-treatment period, the REM latency approached the normal control value (88.5±16.5 minutes as compared with the control value of 100.6 ±20.2 minutes); the difference in REM latency was however not significant (p=0.085).

**Table 3:** Sleep (REM latency) and cognitive function test measures in the study groups

	Index cases		
		
Parameter	Before treatment	After treatment	Normal controls
Sleep measure (REM latency) (in minutes; mean±SD)	61.4±34.7	88.5±16.5	100.6±20.2
Card Sort Test (Mean±SD)			
% correct response	41.3±10.8	49.8±11.0	58.0±5.6
Perseverative error response	23.9± 20.3	16.2±9.2	12±7.2
Stroop Test (% correct response)	74.9±10.0	79. 5±9.9	92.5±2.0
VISCPT (% correct response)	71.7±21.1	79.1±10.1	94.5±2.0
Spatial Memory Test (% correct response)	94.7±4.2	98.2±2.1	99.8±0.5

VISCPT: Visual Continuous Performance Task

The various neurocognitive test measures showed significant global impairment in patients with schizophrenia as compared to normal controls (p values at 0.01 to 0.05). In the pretreatment phase, the percentage correct response and perseverative error response with the Card Sort Test in patients with schizophrenia were 41.3±10.8 and 23.9±20.3, respectively. These were significantly lower (p=0.05) than scores of those in the control group (58.0±5.6 and 12.0±7.2, respectively). The percentage correct response in Spatial Memory Test, VISCPT and Stroop Test was 94.5±2, and 92.5±2 in the control group (p<0.05). Though there were changes in these values in patients with schizophrenia after treatment, these changes were not robust enough to reach any significant level. The results of the tests showed that the patient group had significant impairment in neurocognitive abilities, both before and after treatment.

## DISCUSSION

Patients with schizophrenia demonstrated neuropsychological deficits on tests of attention, memory and abstract reasoning. The patients demonstrated modest evidence of perseverative response on the Card Sort Test, which is interesting and merits consideration. It might support the hypofrontality hypothesis of schizophrenia.

The most significant finding of the study was the positive correlation between the severity of negative symptoms and neurocognitive deficits. It is possible that putatively frontally mediated perseverative tendencies among patients with schizophrenia are due to the transient withdrawal of patients from the stabilizing effects of antipsychotic medication.^24^ In the study by Weinberger *et al.,*^27^ long-term hospitalized patients with chronic schizophrenia who had not been taking medications for 4 weeks before testing, showed similar responses. Improvement in neuropsychological performance might be explained by the fact that antipsychotic medications have a stabilizing effect on the brain's mesolimbic frontal dopamine system. This hypothesis is consistent with the findings of previous studies^28^–^30^ which have shown that initial deficits in information processing during the acute phase tend to normalize over time with appropriate medication. However, there have been arguments against this interpretation. Berman *et al.*^31^ ascribed the refractoriness of WCST deficits in patients with schizophrenia to intentional and medication factors. On comparing groups of neuroleptic-treated and neuroleptic-free patients with schizophrenia and normal controls on the WCST result, it was found that WCST results were not affected by the medication status of the groups. The authors concluded that ‘state factors’ such as attention, effort or global psychosis do not account for the observed pattern of results.

The most confusing result that we came across in our study is the significantly shortened REM latency, which apparently 'normalizes' after antipsychotic treatment and the positive correlation of this with the severity of negative symptoms and cognitive deficits. There have been studies^32^–^34^ which hypothesized that cholinergic hyperactivity (muscarinic supersensitivity) might be responsible for the shortened REM latency. Similarly, a positive correlation found between the severity of negative symptoms and shortened REM latency in different studies,^35^–^37^ prompted these authors to hypothesize the cholinergic mechanism of negative schizophrenic symptoms. Our study also showed a similar correlation between the severity of negative symptoms, REM latency and severity of cognitive deficits. So we can hypothesize that the acetylcholine deficit model of dementia cannot be applied to schizophrenic dementia, rather a hypercholinergic state results, which warrants anticholinergic medication as a treatment option for the negative symptoms of schizophrenia.Further evidence of the hypercholinergic state in chronic, predominantly negative, schizophrenia is evident from the abuse of anticholinergic medication in patients with schizophrenia.
